# Hematological parameters and prevalence of anemia in white and British Indian vegetarians and nonvegetarians in the UK Biobank

**DOI:** 10.1093/ajcn/nqz072

**Published:** 2019-06-13

**Authors:** Tammy Y N Tong, Timothy J Key, Kezia Gaitskell, Timothy J Green, Wenji Guo, Thomas A Sanders, Kathryn E Bradbury

**Affiliations:** 1 Cancer Epidemiology Unit, Nuffield Department of Population Health, Oxford, United Kingdom; 2 Nuffield Division of Clinical Laboratory Sciences, Radcliffe Department of Medicine, University of Oxford, Oxford, United Kingdom; 3 Discipline of Paediatrics and Reproductive Health, School of Medicine, University of Adelaide, Adelaide, Australia; 4 Department of Nutritional Sciences, King's College London, London, United Kingdom; 5 National Institute for Health Innovation, School of Population Health, Faculty of Medical and Health Sciences, University of Auckland, Auckland, New Zealand

**Keywords:** UK Biobank, vegetarian, vegan, hematology, blood count, anemia, ethnicity

## Abstract

**Background:**

There may be differences in hematological parameters between meat-eaters and vegetarians.

**Objective:**

The aim of this study was to perform cross-sectional analyses of hematological parameters by diet group in a large cohort in the United Kingdom.

**Methods:**

A complete blood count was carried out in all UK Biobank participants at recruitment (2006–2010). We examined hemoglobin, red and white blood cell counts, and platelet counts and volume in regular meat eaters (>3 times/wk of red/processed meat consumption, *n* = 212,831), low meat eaters (*n* = 213,092), poultry eaters (*n* = 4815), fish eaters (*n* = 10,042), vegetarians (*n* = 6548), and vegans (*n* = 398) of white ethnicity and meat eaters (*n* = 3875) and vegetarians (*n* = 1362) of British Indian ethnicity.

**Results:**

In both white and British Indian populations, compared with regular meat eaters (or meat eaters in Indians), the other diet groups had up to 3.7% lower age-adjusted hemoglobin concentrations (difference not significant in white vegan women) and were generally more likely to have anemia (e.g., 8.7% of regular meat eaters compared with 12.8% of vegetarians in white premenopausal women; *P* < 0.05 after Bonferroni correction). In the white population, compared with regular meat eaters, all other diet groups had lower age- and sex-adjusted total white cells, neutrophils, lymphocytes, monocytes, and eosinophils (*P*-heterogeneity < 0.001 for all), but basophil counts were similar across diet groups; in British Indians, there was no significant difference in any of the white blood cell counts by diet group. Compared with white regular meat eaters, the low meat eaters, poultry eaters, fish eaters, and vegans had significantly lower platelet counts and higher platelet volume, whereas vegetarians had higher counts and lower volume. Compared with British Indian meat eaters, vegetarians had higher platelet count and lower volume.

**Conclusions:**

In the UK Biobank, people with low or no red meat intake generally had lower hemoglobin concentrations and were slightly more likely to be anemic. The lower white blood cell counts observed in low and non-meat eaters, and differences in mean platelet counts and volume between diet groups, warrant further investigation. This observational study was registered at http://www.isrctn.com/ as ISRCTN10125697.

## Introduction

Vegetarian and vegan diets are associated with lower BMI, blood pressure, and blood cholesterol concentrations (1–3). At the same time, the long-term exclusion of animal foods may lead to inadequate intakes of some essential nutrients that are not easily obtained from plant sources, especially without fortification (4, 5), and this may in turn affect hematological parameters. For example, red meat is a good source of heme iron, and poultry and fish also contain heme iron, which is more bioavailable than plant sources of iron; therefore, lack of meat or fish consumption may increase risk of iron-deficiency anemia (6). Indeed, several small studies reported that vegetarians and vegans had higher rates of anemia and lower hemoglobin or red blood cell counts compared with nonvegetarians (7–11). Vitamin B-12 is only present in animal foods; vegetarians, or vegans who exclude all animal foods from their diet, generally have a higher prevalence of vitamin B-12 deficiency (4, 12). Vitamin B-12 has a crucial role in various cellular processes, including the maturation of red blood cells (13), and may play a role in the platelet life cycle (14–16). Intake of dietary protein, or specific amino acids, which may be low in vegetarian diets (17), may also have a role in supporting the immune system, including production or activation of white blood cells (18, 19). Other nutrients, such as zinc, vitamin A, and riboflavin, which are less bioavailable in plant-based diets (5, 20), have also been linked to blood cell or hemoglobin production (21–25).

There is limited robust evidence on the influence of vegetarian diets on hematological parameters, which might be reflective of anemia or immune status. Here, we examine the associations between varying degrees of animal source food exclusion and hematological indices in a large population-based cohort of ∼500,000 participants in the United Kingdom who self-identified as white British or British Indians.

## Methods

### Study design and participants

The UK Biobank is a prospective cohort of >500,000 people aged 40–69 y, who were recruited in 2006–2010 across the United Kingdom (26). The scientific rationale and design of the UK Biobank study have been described in detail elsewhere (27). In brief, people who lived within traveling distance (∼25 km) of 1 of the 22 assessment centers across England, Wales, and Scotland were identified from National Health Service registers and invited to participate in the study. Permission for access to patient records for recruitment was approved by the Patient Information Advisory Group (now the National Information Governance Board for Health and Social Care) in England and Wales and by the Community Health Index Advisory Group in Scotland. Overall, ∼5.5% of the invitees attended a baseline visit (28) during which they gave informed consent to participate in UK Biobank using a signature capture device and completed a touch-screen questionnaire that asked about sociodemographic characteristics, lifestyle exposures [including diet, supplement use (e.g., formulations containing B vitamins, folic acid, multiple micronutrients, or iron), and smoking status], and general health and medical history. All participants also completed a computer-assisted personal interview and had physical measurements and blood samples taken. In addition to a self-administered questionnaire, additional dietary information was collected using a Web-based 24-h dietary assessment tool (29), which was administered ≤5 times in a large subsample of participants (∼210,000). This study was registered at http://www.isrctn.com/ as ISRCTN10125697.

### Ethnicity classification

On the touch-screen questionnaire, participants were asked to identify their ethnicity from options of “White,” “Mixed,” “Asian or Asian British,” “Black or Black British,” “Chinese,” “Other ethnic group,” “Do not know,” or “Prefer not to answer.” Participants were included for analyses if they self-identified as “white” or as “Asian or Asian British” and subsequently as “Indian.” For consistency, participants are subsequently referred to as “white” or “British Indian.” The white population was included because it made up the majority of the UK Biobank population (∼94%), and the British Indian population was included due to the large proportion of vegetarians in this population group (24.6% compared with 1.7% in the overall cohort). The number of vegetarians in the other ethnic groups was small; therefore, other ethnic groups were excluded from these analyses.

### Diet group classification

To determine the classification of diet groups, we used methods previously described (1, 30). Briefly, participants were asked their frequency of consumption of processed meat, beef, lamb or mutton, pork, poultry (e.g., chicken or turkey), oily fish, other types of fish, eggs or foods containing eggs, and dairy products, in 6 categories of frequency ranging from “Never” to “Once or more daily.” Based on these questions, 6 diet groups were defined for the white British population: regular meat eaters (red and processed meat consumption >3 times/wk), low meat eaters (red and processed meat consumption ≤3 times/wk), poultry eaters (participants who ate poultry but no red or processed meat, regardless of whether they ate fish, dairy products, or eggs), fish eaters (participants who ate fish but no red or processed meat or poultry), vegetarians (participants who did not eat meat, poultry, or fish), and vegans (participants who further excluded dairy products and eggs). Two diet groups were defined for the British Indian population: meat eaters (ate any combination of red or processed meat or poultry) and vegetarians (excluding vegans). Information collected from the Web-based 24-h dietary assessment tool was used to estimate food and nutrient intakes (e.g., iron and vitamin B-12) in each diet group, based on *McCance and Widdowson's The Composition of Foods* and its supplements (29, 31).

### Blood measurements and hematological assays

Blood sampling was performed by either a phlebotomist or a nurse in all participants except for a small proportion (0.3%) who declined, were deemed unable to undergo sampling, or where the attempt was abandoned for either technical or health reasons. Nonfasting blood samples were taken from a vein in the inner elbow using an 18-G vacutainer needle and barrel or, if that appeared unsuitable, from a vein on the back of the hand using a 21-G Safety Lok butterfly needle (BD) connected to a vacutainer barrel (27). For hematological assays, blood was collected into a 4-mL EDTA vacutainer and dispatched to the central processing laboratory in temperature-controlled shipping boxes (at 4°C) (32). Complete blood cell counts were conducted using a Coulter Counter (Beckman Coulter), typically within 24 h of blood collection (32, 33). Because the hematological assays were performed throughout a long recruitment period (∼5 y), variation in blood count caused by laboratory drift cannot be ruled out despite efforts in quality control (further details are provided in the **Supplementary Methods**) (32), but any possible variation should be independent of diet group.

### Classification of anemia, low platelet count, and elevated platelet volume

Anemia was defined as hemoglobin concentrations of <130 g/L and <120 g/L for men and women, respectively, based on WHO criteria (34). Anemia severity was classified as mild (hemoglobin 110–129 g/L in men and 110–119 g/L in women), moderate (80–109 g/L in both sexes), and severe (<80 g/L in both sexes). To adjust for the effect of smoking on hemoglobin, when using hemoglobin levels to define anemia, 3 g/L was subtracted for all participants who indicated they were smokers (35), but original values of hemoglobin were used in all other analyses. If anemia was present, it was further defined as microcytic or macrocytic, based on a mean corpuscular volume of <80 fL or >100 fL, respectively (36). Secondarily, we also tested for any differences in the classification by correcting for hemoglobin levels using more detailed categorization of smoking status (<10 cigarettes smoked per day, no adjustment; ≥10 and <20 cigarettes smoked per day, −3 g/L; ≥20 and <40 cigarettes smoked per day, −5 g/L; ≥40 cigarettes smoked per day, −7 g/L; and unknown amount, −3 g/L).

For platelet count and volume, low platelet count was defined as <169.06 × 10^9^ cells/L, and elevated platelet volume was defined as >11.24 fL, both as specified by the manufacturer's reference range (33).

### Statistical analyses

Baseline characteristics of the cohort were tabulated by 6 diet groups in white British participants and by 2 diet groups in British Indian participants. The primary outcomes of this research were blood counts and prevalence of anemia, and the secondary outcomes were prevalence of subtypes of anemia and prevalence of low platelet counts and elevated platelet volume. Linear regressions were modeled to estimate the adjusted mean levels (95% CIs) of each hematological parameter of interest, including hemoglobin, red blood cell count, reticulocyte percentage, immature reticulocyte fraction, total white blood cell count, neutrophils, lymphocytes, monocytes, eosinophils, basophils, platelet count, and platelet volume.

To estimate mean levels of hemoglobin, red blood cell count, reticulocyte percentage, and immature reticulocyte fraction, the regression model was stratified by sex and menopausal status (men, premenopausal women, and postmenopausal women) and adjusted for age of recruitment (5-y age groups from <45, 45–49, 50–54, 55–59, 60–64, and ≥65 y). We additionally adjusted for smoking status (never, previous, current <15 cigarettes per day, current ≥15 cigarettes per day, and unknown) in a second model. There was little difference in reticulocyte percentage and immature reticulocyte fraction by sex and menopausal status; thus, we combined the 3 groups and adjusted for sex in the regression model for our main results and presented the stratified results secondarily.

To estimate mean levels of white cells (total white blood cell count, neutrophils, lymphocytes, monocytes, eosinophils, and basophils) and platelets (platelet count and platelet volume), the regression model was adjusted for age and sex and subsequently for smoking status. In addition, as a third model, we restricted the analysis to participants who answered “No” to the question “Do you have any long-standing illness, disability, or infirmity?” to remove the possible confounding effect of chronic illnesses on white cell or platelet count.

For classification of anemia, low platelet count, and elevated platelet volume, numbers and percentages of people in each diet group were reported as observed, based on criteria as previously described. For anemia, we additionally restricted the analyses to people who reported no iron or B vitamins supplement use.

For each baseline characteristic and each hematological parameter of interest, post hoc pairwise comparisons based on linear regression models were used to test for significant differences between diet groups. For the white population, Bonferroni correction for multiple comparisons between the 6 diet groups was applied, and results reported here represent significant differences after Bonferroni correction, and by using regular meat eaters as the reference group, unless otherwise stated. All statistical analyses were performed using Stata release 15.1 (StataCorp), and 2-sided *P* values <0.05 were considered significant.

## Results

After excluding participants who had no hematological data (*n* = 24,336), reported other or unknown ethnicities (*n* = 21,851), or did not answer a sufficient number of questions to be classified into a diet group (*n* = 3393), 447,726 white British and 5237 British Indian participants were included in this analysis. Of the white British participants, 212,831 were classified as regular meat eaters, 213,092 as low meat eaters, 4815 as poultry eaters, 10,042 as fish eaters, 6548 as vegetarians, and 398 as vegans. Of the British Indian participants, 3875 were classified as meat eaters and 1362 as vegetarians. A participant flowchart of the inclusion and exclusion criteria of this study is shown in **Supplemental Figure 1**.

### Participant characteristics

Characteristics of UK Biobank participants are shown in **Tables 1** and **2**. In the white population, low or non-meat eaters (fish eaters, vegetarians, and vegans) were more likely to be women, and non-meat eaters were younger. Except for vegans, low and non-meat eaters were less likely to report current smoking and the presence of long-standing illness. Non-meat eaters had a higher reported use of some specified supplements (formulations containing B vitamins, folic acid, multiple micronutrients, or iron), and low and non-meat eaters had on average higher total iron intake from foods (but lower or no iron intake from red or processed meat) and lower vitamin B-12 intake from foods (especially in vegetarians and vegans). Regarding the British Indian participants, vegetarians were slightly older and more likely to be women. They were less likely to report current smoking, more likely to report specified supplement use [formulations containing B vitamins, folic acid, multiple micronutrients, or iron (significant in women only)], and had lower mean vitamin B-12 intake from foods.

**TABLE 1:** Baseline characteristics of white British participants by diet group in the UK Biobank^1^

	Meat eaters						
Characteristics	Regular consumption (>3 times/wk) (max *n* = 212,831)^2^	Low consumption (≤ 3 times/wk) (max *n* = 213,092)^2^	Poultry eaters (max *n* = 4815)	Fish eaters (max *n* = 10,042)	Vegetarians (max *n* = 6548)	Vegans (max *n* = 398)	*P*-het^3^
Age, y	56.8 ± 8.1^a^	56.9 ± 7.9^b^	56.7 ± 8.0^a,b^	54.2 ± 8.0^c^	52.8 ± 7.9^d^	54.3 ± 7.9^c^	<0.001
Women, *n* (%)	91,398 (42.9)^a^	135,635 (63.7)^b^	3741 (77.7)^c^	7253 (72.2)^d^	4418 (67.5)^e^	232 (58.3)^b^	<0.001
Premenopausal	20,791 (22.7)	29,570 (21.8)	810 (21.7)	2287 (31.5)	1636 (37.0)	76 (32.8)	
Postmenopausal	67,145 (73.5)^a^	101,184 (74.6)^b^	2797 (74.8)^a,b^	4643 (64.0)^c^	2583 (58.5)^d^	149 (64.2)^a,b,c,d^	<0.001
Smoking status, *n* (%)							
Previous	76,402 (36.0)	74,826 (35.2)	1675 (34.9)	3705 (37.0)	2213 (33.9)	157 (39.5)	
Current	25,980 (12.2)^a^	18,852 (8.9)^b^	360 (7.5)^c^	719 (7.2)^b,c^	512 (7.8)^c^	31 (7.8)^a,b,c^	<0.001
Has a long-standing illness, *n* (%)	71,900 (34.6)h^a^	63,876 (30.7)^b^	1498 (31.8)^b^	2703 (27.5)^c^	1835 (28.7)^c,d^	140 (35.8)^a,b,d^	<0.001
Regular supplement user, *n* (%)							
B vitamins	7541 (3.5)^a^	9369 (4.4)^b^	449 (9.3)^c^	739 (7.4)^d^	541 (8.3)^c,d^	76 (19.1)^e^	<0.001
Folic acid	3985 (1.9)^a^	4883 (2.3)^b^	214 (4.4)^c^	335 (3.3)^d^	199 (3.0)^d^	19 (4.8)^c,d^	<0.001
Multiple micronutrients	40,911 (19.2)^a^	48,649 (22.8)^b^	1561 (32.4)^c,d^	3122 (31.1)^c^	2248 (34.3)^d,e^	155 (38.9)^e^	<0.001
Iron, in men	2333 (1.9)^a^	1615 (2.1)^a^	50 (4.7)^b^	160 (5.7)^b,c^	134 (6.3)^c^	15 (9.0)^c^	<0.001
Iron, in premenopausal women	1226 (5.9)^a^	1888 (6.4)^a^	76 (9.4)^b^	300 (13.1)^c^	236 (14.4)^c^	7 (9.2)^a,b,c^	<0.001
Iron, in postmenopausal women	1667 (2.5)^e^	2749 (2.7)^e^	152 (5.4)^d^	327 (7.0)^c^	215 (8.3)^b^	23 (15.4)^a^	<0.001
Mean dietary iron intake, mg/d (95% CI)^4^	13.7 (13.7, 13.8)^a^	13.5 (13.5, 13.5)^b^	13.9 (13.7, 14.1)^b^	14.6 (14.5, 14.7)^c^	14.6 (14.4, 14.7)^c^	16.9 (16.4, 17.4)^d^	<0.001
From red or processed meat^4^	1.42 (1.41, 1.43)^a^	1.02 (1.01, 1.03)^b^	—	—	—	—	<0.001
From poultry and fish^4^	0.56 (0.55, 0.56)^a^	0.60 (0.59, 0.60)^b^	0.74 (0.71, 0.76)^c^	0.48 (0.46, 0.49)^d^	—	—	<0.001
Mean dietary vitamin B-12 intake, µg/d (95% CI)^4^	6.74 (6.71, 6.77)^a^	6.48 (6.45, 6.51)^b^	6.42 (6.23, 6.60)^b^	6.03 (5.91, 6.14)^c^	2.96 (2.82, 3.10)^d^	0.88 (0.31, 1.45)^e^	<0.001

^1^Values are means ± SDs unless otherwise indicated; *n* = 447,726. Groups that do not share a superscript letter were significantly different at the 5% level from post hoc pairwise comparisons based on linear regression models and after Bonferroni correction for multiple comparisons. max, maximum.

2 Includes participants who consume any red or processed meat (beef, lamb, pork, and processed meat), regardless of whether they consume poultry, fish, dairy, or eggs. Cutoffs of regular and low consumption were determined based on consumption of red and processed meat as reported on the touch-screen questionnaire.

3 Represents *P* for heterogeneity across the six diet groups, estimated by regressing each baseline characteristic against diet group.

4 Estimated based on a subsample who completed ≥1 Web-based 24-h dietary assessment and based on intakes from food sources only. Assessments were averaged for participants who completed >1 assessment. The numbers of white British participants who completed ≥1 dietary assessments were as follows: 87,525 regular meat eaters, 93,513 low meat eaters, 2165 poultry eaters, 5486 fish eaters, 3745 vegetarians, and 232 vegans. Estimates were adjusted for age at recruitment (<45, 45–49, 50–54, 55–59, 60–64, and ≥65 y).

**TABLE 2:** Baseline characteristics of British Indian participants by diet group in the UK Biobank^1^

Characteristics	Meat eaters (max *n* = 3875)	Vegetarians (max *n* = 1362)	*P*-het^2^
Age, y	53.7 ± 8.4	55.0 ± 7.9	<0.001
Women, *n* (%)	1621 (41.8)	877 (64.4)	<0.001
Premenopausal	565 (34.9)	229 (26.1)	
Postmenopausal	980 (60.5)	615 (70.1)	<0.001
Smoking status, *n* (%)			
Previous	530 (13.8)	87 (6.4)	
Current	350 (9.1)	30 (2.2)	<0.001
Has a long-standing illness, *n* (%)	1170 (31.5)	385 (29.5)	0.18
Regular supplement user, *n* (%)			
Vitamin B	198 (5.1)	89 (6.5)	0.047
Folic acid or folate	133 (3.4)	67 (4.9)	0.014
Multivitamins ± minerals	971 (25.1)	387 (28.4)	0.015
Iron, in men	82 (3.6)	23 (4.7)	0.25
Iron, in premenopausal women	79 (14.0)	46 (20.1)	0.032
Iron, in postmenopausal women	77 (13.6)	38 (16.6)	0.033
Mean dietary iron intake, mg/d (95% CI)^3^	11.8 (11.6, 12.1)	11.6 (11.1, 12.1)	0.34
From red or processed meat^3^	0.72 (0.65, 0.78)	—	—
From poultry and fish^3^	0.60 (0.56, 0.63)	—	—
Mean dietary vitamin B-12 intake, µg/d (95% CI)^3^	5.20 (4.95, 5.44)	2.55 (2.10, 3.00)	<0.001

1 Values are means ± SDs unless otherwise indicated; *n* = 5237. max, maximum.

2 Represents *P* for heterogeneity across the 2 diet groups, estimated by regressing each baseline characteristic against diet group.

3 Estimated based on a subsample who completed ≥1 Web-based 24-h dietary assessment and based on intakes from food sources only. Assessments were averaged for participants who completed >1 assessment. The numbers of British Indian participants who completed ≥1 dietary assessments were as follows: 1333 meat eaters, 397 vegetarians, and 248 vegans. Estimates were adjusted for age at recruitment (<45, 45–49, 50–54, 55–59, 60–64, and ≥65 y).

### Hemoglobin levels, red blood cell counts, and prevalence of anemia

**FIGURE 1:**
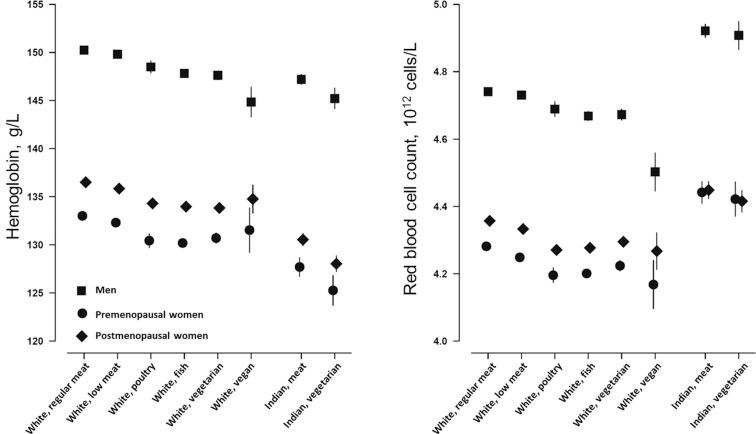
Hemoglobin concentration and red blood cell count by diet group and ethnicity in the UK Biobank. Point estimates represent adjusted mean levels (95% CIs), estimated based on linear regression models. All estimates were adjusted for age at recruitment (<45, 45–49, 50–54, 55–59, 60–64, and ≥65 y) and smoking (never, previous, current <15 cigarettes/d, current ≥15 cigarettes/d, and unknown). Total numbers of men, premenopausal women, and postmenopausal women, respectively, in the diet groups were as follows: white regular meat eaters: 121,433, 20,791, 67,145; white low meat eaters: 77,457, 29,570, 101,184; white poultry eaters: 1074, 810, 2797; white fish eaters: 2789, 2287, 4643; white vegetarians: 2130, 1636, 2583; white vegans: 166, 76, 149; Indian meat eaters: 2254, 565, 980; Indian vegetarians: 485, 229, 615. *P* for heterogeneity across the diet groups (stratified by ethnicity and estimated by regressing each variable against diet group) was 0.01 for hemoglobin in British Indian premenopausal women, >0.5 for red blood cell count in all British Indians, and <0.001 for all other variables.

**FIGURE 2:**
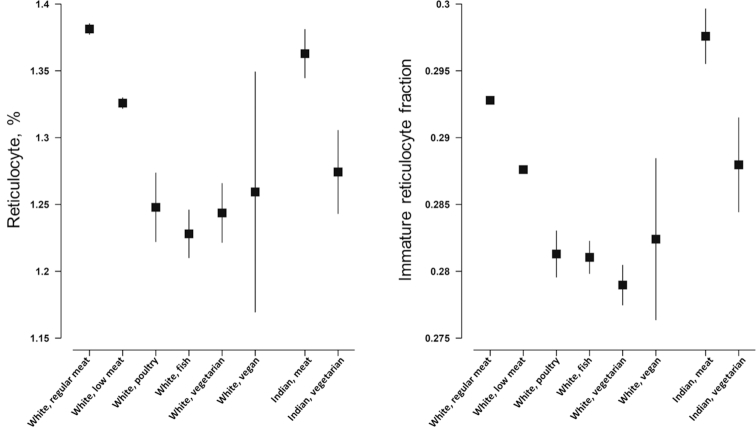
Reticulocyte percentage and immature reticulocyte fraction by diet group and ethnicity in the UK Biobank. Point estimates represent adjusted mean levels (95% CIs), estimated based on linear regression models. All estimates were adjusted for age at recruitment (<45, 45–49, 50–54, 55–59, 60–64, and ≥65 y), sex, and smoking (never, previous, current <15 cigarettes/d, current ≥15 cigarettes/d, and unknown). Total numbers of participants in the diet groups were as follows: white regular meat eaters, 212,831; white low meat eaters, 213,092; white poultry eaters, 4815; white fish eaters, 10,042; white vegetarians, 6548; white vegans, 398; Indian meat eaters, 3875; and Indian vegetarians, 1362. *P* for heterogeneity across the diet groups (stratified by ethnicity and estimated by regressing each variable against diet group) was >0.2 for reticulocyte percentage and immature reticulocyte fraction in British Indian premenopausal women and <0.001 for all other variables.

Mean hemoglobin concentration, red blood cell count, reticulocyte percentage, and immature reticulocyte fraction are shown in **Figures 1** and **2** and **Supplemental Tables 1**–**6**. In white British participants, compared with regular meat eaters, all other diet groups had lower hemoglobin concentrations [e.g., 150.3 g/L (95% CI: 150.2, 150.3 g/L) in regular meat-eating men compared with 144.8 g/L (95% CI: 143.2, 146.3 g/L) in vegan men], but the difference was not statistically significant in vegan women (Figure 1 and Supplemental Tables 1–6). All other diet groups also had lower red blood cell counts, lower reticulocyte percentages (not significant in vegans), and lower immature reticulocyte fraction (not significant in vegan men and premenopausal women who were low meat eaters, poultry eaters, or vegans). In British Indians, vegetarians had lower mean hemoglobin [e.g., 147.2 g/L (95% CI: 146.7, 147.7 g/L) in meat-eating men compared with 145.2 g/L (95% CI: 144.1, 146.2 g/L) in vegetarian men], reticulocyte percentage (not significant in premenopausal women), and immature reticulocyte fraction (not significant in premenopausal women), but similar red blood cell counts. In all populations, results were similar with or without adjustment for smoking.

Numbers and proportions of people with anemia are reported in **Tables 3** and **4**. In white men, the proportion of people with anemia was significantly higher in fish eaters compared with regular meat eaters (3.9% compared with 2.9%, respectively), and although proportions with anemia were also higher in poultry eaters, vegetarians, and vegans, the difference was not statistically significant (Table 3). In both white premenopausal and postmenopausal women, compared with regular meat eaters, low meat eaters, poultry eaters, fish eaters, and vegetarians (12.8% of vegetarians compared with 8.7% of regular meat eaters in premenopausal women and 5.8% of vegetarians compared with 3.4% of regular meat eaters in postmenopausal women) were more likely to have anemia. In British Indians, vegetarian men and postmenopausal women were more likely to have anemia compared with meat eaters (12.6% of vegetarian men compared with 7.6% of meat eaters and 19.2% of vegetarian postmenopausal women compared with 13.3% of meat eaters), but there was no statistically significant difference in premenopausal women (26.6% of vegetarian premenopausal women compared with 20.5% of meat eaters) (Table 4). Results were similar when we restricted the analyses to participants who did not report taking iron or B vitamin supplements and also when we applied more detailed correction for smoking; proportions of participants with microcytic or macrocytic anemia were small in all subgroups (**Supplemental Tables 7** and **8**).

**TABLE 3:** Anemia prevalence in white British participants by diet group in the UK Biobank^1^

	Meat eaters						
Classification^2^	Regular consumption (>3 times/wk) (total *n* = 212,831)^3^	Low consumption (≤3 times/wk) (total *n* = 213,092)^3^	Poultry eaters (total *n* = 4815)	Fish eaters (total *n* = 10,042)	Vegetarians (total *n* = 6548)	Vegans (total *n* = 398)	*P*-het^4^
Anemia in men, *n* (%)	3517 (2.9)^a^	2200 (2.8)^a^	43 (4.0)^a,b^	108 (3.9)^b^	83 (3.9)^a,b^	11 (6.6)^a,b^	<0.001
Mild	3239 (2.7)	2049 (2.6)	38 (3.5)	104 (3.7)	78 (3.7)	9 (5.4)	
Moderate	261 (0.2)	142 (0.2)	5 (0.5)	4 (0.1)	5 (0.2)	1 (0.6)	
Severe	17 (0.0)^a^	9 (0.0)^a^	0 (0.0)^a,b^	0 (0.0)^a^	0 (0.0)^a,b^	1 (0.6)^b^	<0.001
Anemia in premenopausal women, *n* (%)	1804 (8.7)^a^	2888 (9.8)^b^	105 (13.0)^c^	309 (13.5)^c^	209 (12.8)^c^	6 (7.9)^a,b,c^	<0.001
Mild	1346 (6.5)	2097 (7.1)	81 (10.0)	230 (10.1)	146 (8.9)	4 (5.3)	
Moderate	442 (2.1)	766 (2.6)	23 (2.8)	75 (3.3)	63 (3.9)	0 (0.0)	
Severe	16 (0.1)^a^	25 (0.1)^b^	1 (0.1)^b,c^	4 (0.2)^c^	0 (0.0)^c^	2 (2.6)^a,b,c^	<0.001
Anemia in postmenopausal women, *n* (%)	2300 (3.4)^a^	3838 (3.8)^b^	154 (5.5)^c^	248 (5.3)^c^	149 (5.8)^c^	6 (4.0)^a,b,c^	<0.001
Mild	1974 (2.9)	3331 (3.3)	140 (5.0)	218 (4.7)	129 (5.0)	5 (3.4)	
Moderate	315 (0.5)	494 (0.5)	13 (0.5)	30 (0.6)	20 (0.8)	0 (0.0)	
Severe	11 (0.0)^a^	13 (0.0)^b^	1 (0.0)^c^	0 (0.0)^c^	0 (0.0)^c^	1 (0.7)^a,b,c^	<0.001

1 *n* = 447,726. Groups that do not share a superscript letter were significantly different at the 5% level from post hoc pairwise comparisons based on linear regression models and after Bonferroni correction for multiple comparisons.

2 Anemia is defined as hemoglobin <130 g/L for men and <120 g/L for women. Mild anemia, hemoglobin 110–129 g/L in men and 110–119 g/L in women; moderate anemia, hemoglobin 80–109 g/L (both sexes); and severe anemia, hemoglobin <80 g/L (both sexes). For defining anemia and all subtypes of anemia, hemoglobin is adjusted by −3 g/L in current smokers.

3 Includes participants who consume any red or processed meat (beef, lamb, pork, and processed meat), regardless of whether they consume poultry, fish, dairy, or eggs. Cutoffs of regular and low consumption were determined based on consumption of red and processed meat as reported on the touch-screen questionnaire.

4 Represents *P* for heterogeneity across the 6 diet groups, estimated by regressing each row variable against diet group.

**TABLE 4:** Anemia prevalence in British Indian participants by diet group in the UK Biobank^1^

Classification	Meat eaters (total *n* = 3875)	Vegetarians (total *n* = 1362)	*P*-het^2^
Anemia in men, *n* (%)	172 (7.6)	61 (12.6)	<0.001
Mild	157 (7.0)	59 (12.2)	
Moderate	15 (0.7)	2 (0.4)	
Severe	0 (0.0)	0 (0.0)	0.002
Anemia in premenopausal women, *n* (%)	116 (20.5)	61 (26.6)	0.061
Mild	80 (14.2)	38 (16.6)	
Moderate	35 (6.2)	23 (10.0)	
Severe	1 (0.2)	0 (0.0)	0.042
Anemia in postmenopausal women, *n* (%)	130 (13.3)	118 (19.2)	0.002
Mild	106 (10.8)	92 (15.0)	
Moderate	24 (2.4)	24 (3.9)	
Severe	0 (0.0)	2 (0.3)	<0.001

1 *n* = 5237. Anemia is defined as hemoglobin <130 g/L for men and <120 g/L for women. Mild anemia, hemoglobin 110–129 g/L in men and 110–119 g/L in women; moderate anemia, hemoglobin 80–109 g/L (both sexes); and severe anemia, hemoglobin <80 g/L (both sexes). For defining anemia and all subtypes of anemia, hemoglobin is adjusted by −3 g/L in current smokers.

2 Represents *P* for heterogeneity across the 2 diet groups, estimated by regressing each row variable against diet group.

### White blood cell count

**FIGURE 3:**
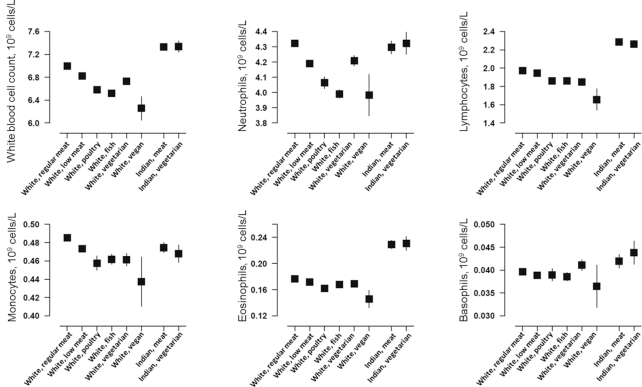
White blood cell counts by diet group and ethnicity in the UK Biobank. Point estimates represent adjusted mean levels (95% CIs), estimated based on linear regression models. All estimates were adjusted for age at recruitment (<45, 45–49, 50–54, 55–59, 60–64, and ≥65 y), sex, and smoking (never, previous, current <15 cigarettes/d, current ≥15 cigarettes/d, and unknown). Total numbers of participants in the diet groups were as follows: white regular meat eaters, 212,831; white low meat eaters, 213,092; white poultry eaters, 4815; white fish eaters, 10,042; white vegetarians, 6548; white vegans, 398; Indian meat eaters, 3875; and Indian vegetarians, 1362. *P* for heterogeneity across the diet groups (stratified by ethnicity and estimated by regressing each variable against diet group) was >0.1 for all variables in British Indians and <0.001 for all variables in white British participants.

Total and specific (neutrophils, lymphocytes, monocytes, eosinophils, and basophils) white blood cell counts are shown by diet group in **Figure 3** and **Supplemental Tables 9** and **10**. In the white population, compared with regular meat eaters, all other diet groups had lower mean counts of total white cells [e.g., 7.02 × 10^9^ cells/L (95% CI: 7.01, 7.03 × 10^9^ cells/L) in regular meat eaters compared with 6.22 × 10^9^ cells/L (95% CI: 6.01, 6.43 × 10^9^ cells/L) in vegans], neutrophils, lymphocytes, monocytes, and eosinophils; basophil counts appeared similar across diet groups, although counts were low overall. In the Indian population, there was no significant difference in any of the white blood cell counts between meat eaters and vegetarians. Results were similar with or without adjustment for smoking and when including only people who reported having no long-standing illness (*n* = 295,590 in white British participants; *n* = 3465 in British Indian participants).

### Platelet count and volume

**FIGURE 4:**
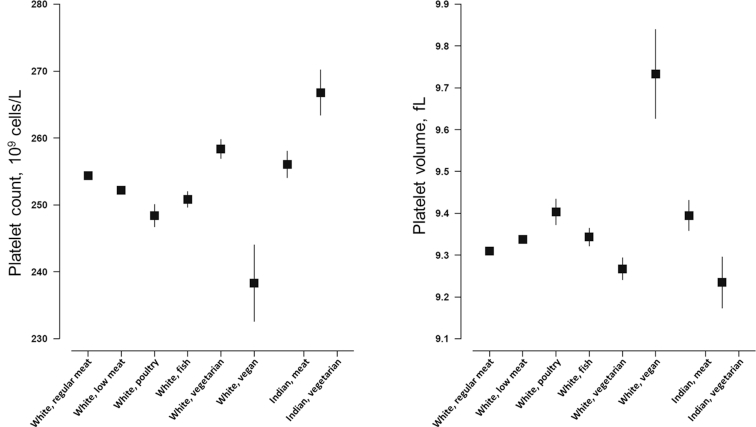
Platelet count and volume by diet group and ethnicity in the UK Biobank. Point estimates represent adjusted mean levels (95% CIs), estimated based on linear regression models. All estimates were adjusted for age at recruitment (<45, 45–49, 50–54, 55–59, 60–64, and ≥65 y), sex, and smoking (never, previous, current <15 cigarettes/d, current ≥15 cigarettes/d, and unknown). Total numbers of participants in the diet groups were as follows: white regular meat eaters, 212,831; white low meat eaters, 213,092; white poultry eaters, 4815; white fish eaters, 10,042; white vegetarians, 6548; white vegans, 398; Indian meat eaters, 3875; and Indian vegetarians, 1362. *P* for heterogeneity across the diet groups (stratified by ethnicity and estimated by regressing each variable against diet group) was <0.001 for all variables.

Mean levels of platelet count and platelet volume are plotted in **Figure 4** and shown in Supplemental Tables 9 and 10. Compared with white regular meat eaters (mean: 254.5 × 10^9^ cells/L; 95% CI: 254.2, 254.7 × 10^9^ cells/L), the low meat eaters, poultry eaters, fish eaters, and vegans (mean: 238.2 × 10^9^ cells/L; 95% CI: 232.5, 243.9 × 10^9^ cells/L) had lower mean platelet counts, whereas vegetarians had higher counts (mean: 258.2 × 10^9^ cells/L; 95% CI: 256.8, 259.7 × 10^9^ cells/L). On the other hand, vegetarians had lower mean platelet volume (mean: 9.27 fL; 95% CI: 9.24, 9.29 fL), whereas the other diet groups, especially vegans (mean: 9.73 fL; 95% CI: 9.63, 9.84 fL), had higher platelet volumes compared with regular meat eaters (mean: 9.31 fL; 95% CI: 9.31, 9.32 fL). In British Indians, vegetarians had higher mean platelet count (mean: 266.7 × 10^9^ cells/L; 95% CI: 263.3, 270.0 × 10^9^ cells/L) but lower mean platelet volume (mean: 9.24 fL; 95% CI: 9.18, 9.30 fL) compared with meat eaters (mean: 256.1 × 10^9^ cells/L; 95% CI: 254.1, 258.1 × 10^9^ cells/L; mean: 9.39 fL; 95% CI: 9.36, 9.43 fL). Results were consistent when participants were classified as having low platelet count or elevated platelet volume (Supplemental Tables 7 and 8).

## Discussion

The current study, with >450,000 participants, is the largest study ever conducted to examine hematological parameters by degrees of animal food consumption in white British and British Indian individuals. In the white British population, compared with regular meat eaters, low or non-meat eaters generally had lower hemoglobin concentrations and were more likely to have anemia, and they had lower white blood cell counts. Vegans had lower mean platelet count and higher mean platelet volume, whereas the reverse was true for vegetarians. In British Indians, vegetarians had lower mean hemoglobin, were more likely to be anemic, and had higher platelet count but lower platelet volume compared with meat eaters. In addition to hematological conditions such as anemia or low platelet count, differences in blood count could be related to future risk of diseases (e.g., cardiovascular disease) or mortality (37–39).

Only a few small existing studies (*n* vegetarians <100) have reported on blood cell counts in different diet groups, but our findings corroborate their results. Studies have reported that compared with nonvegetarians, vegetarians or vegans had lower hemoglobin levels (7, 9, 10) or lower red blood cell count (8, 11), but the sample sizes of these studies were too small to accurately assess differences in anemia. Other studies have also reported lower white blood cell counts (including lymphocytes and neutrophils) (7, 14, 40, 41) or platelet counts (41) in vegetarians or vegans compared with nonvegetarians. The only study that included British Indian vegetarians (*n* = 23) found that they had lower hemoglobin but higher platelet count compared with “Caucasian” vegetarians (*n* = 22) (11), which appeared consistent with our results. On the other hand, a few studies reported that compared with nonvegetarians, vegetarians or vegans had similar levels of hemoglobin (42) or white blood cell counts (8), or higher red blood cell counts (43). Overall, because these previous studies were small, they may lack sufficient statistical power to detect potential differences.

Low or non-meat eaters in the UK Biobank would have little or no intake of heme iron (estimated to be ∼40% of iron from meat sources) (44), which is more easily absorbed than non-heme iron (present in plant foods such as cereals, vegetables, pulses, and fruits) (45, 46). Although vegetarian and vegan diets are usually high in vitamin C (47), which enhances iron absorption, plant-based diets also contain significant amounts of phytates and tannins, which inhibit non-heme iron absorption (45). Previous studies showed that vegetarians had lower non-heme and total iron absorption, as well as lower ferritin concentrations, compared with nonvegetarians, despite similar or higher total dietary iron intake (11, 41, 48–50). Given the crucial role of iron in hemoglobin synthesis and red blood cell production (34), it would be expected that compared with regular meat eaters, low or non-meat eaters may have lower levels of hemoglobin and red blood cells and a higher risk of anemia (51). However, as shown in Tables 1 and 2, low or non-meat eaters were more likely to take iron supplements, as well as multivitamins that may contain iron, which could help prevent or correct anemia. For example, anemia in vegans in the UK Biobank seemed to occur mostly in those who were not taking iron supplements, although numbers were too small to make valid conclusions. In addition, zinc is a catalyst in iron metabolism (21) and is less bioavailable in vegetarian diets (5), and low serum zinc levels have been associated with anemia (21, 22). Vegetarians or vegans also tend to have lower intakes of other micronutrients, such as vitamin A or riboflavin (20), which might also have roles in blood cell or hemoglobin production (24, 25).

Another explanation for differences in red blood cell count is that given the crucial role of vitamin B-12 in erythropoiesis, deficiency in this nutrient in vegetarians or vegans may result in red blood cells that do not develop normally and are too large to leave the bone marrow (13, 52, 53), which subsequently manifests as lower counts of reticulocytes and mature red blood cells in these diet groups. For subtypes of anemia, macrocytic anemia can be induced by vitamin B-12 deficiency but can be masked by the simultaneous presence of microcytic anemia in cases of severe iron deficiency or high folate intake (11, 14, 54); previous studies have shown that all 3 exposures are likely in vegetarians (11, 12, 41). Numbers for anemia subtypes were small in this study, however, and serum levels of relevant nutrients and other parameters that would be used in a clinical setting to classify anemia were not available. Hence, it was not possible to determine how these factors affected the distribution of anemia subtypes in different diet groups.

Although anemia might also be caused by β-thalassemia (52), there is no reason to suspect this genetic condition should vary by diet group, but ethnic differences are possible (53). The prevalence of anemia in British Indians in our cohort was much higher than in the corresponding white British groups; iron intake, especially iron from red or processed meat in the meat eaters, and dietary vitamin B-12 intake were also lower in British Indians. However, detailed comparisons of hematological characteristics across different ethnicities are beyond the scope of the current study, which aimed to assess the differences across diet groups.

A high white blood cell count has been proposed as an early marker of inflammation, and in prospective studies it has been associated with a higher risk of chronic diseases or death (37–39). Evidence linking vegetarian diets to risk of chronic inflammation is limited, but some studies suggest that long-term vegetarianism is associated with lower concentrations of other inflammatory biomarkers, such as C-reactive protein (55, 56). On the other hand, a low white blood cell count may also be an indication of an impaired immune system or abnormal bone marrow pathology (57). Vitamin B-12 deficiency is a reversible cause of bone marrow failure (58), and some case reports have suggested a link between vitamin B-12 deficiency (more likely in vegetarians and vegans) and pancytopenia (low counts of red blood cells, white blood cells, and platelets) (59, 60). Differences in intake of other nutrients between the diet groups might also explain the differences in white blood cell count. Some evidence, largely from in vitro or animal studies, suggests that dietary proteins or specific amino acids might be integral to proper functioning of the immune system, including the production of blood cells (18, 19, 61, 62). Parallel evidence from large-scale human studies is lacking, but previous studies do show differences in dietary and serum amino acids between different diet groups (17). Alternatively, zinc deficiency has also been linked to impaired growth and functioning of immune cells (23). Note that although there were differences between the diet groups, all groups had mean white blood cell counts within the normal range (38, 57).

In addition to their role in blood clotting, platelets are also believed to be involved in chronic inflammation, via interactions with endothelial cells and white blood cells (63, 64); hence, it might be reasonable that diet groups with a low white blood cell count should also have a low platelet count. Alternatively, due to the relatively high vitamin B-12 content in platelets compared to red blood cells (15), it has been suggested that vitamin B-12 has a prominent role in the platelet cell life cycle, and low platelet count and large platelet volume can be a symptom of vitamin B-12 deficiency (14). Studies have documented that low platelet counts in vitamin B-12-deficient patients have responded to vitamin B-12 therapy (16, 65). This fits with the observations of low platelet count and large platelet volume in the vegans in this study, but it does not explain the reverse pattern in vegetarians, who also have relatively low vitamin B-12 intakes.

Strengths of this study include the large sample size of ∼450,000 white and 5000 British Indians in the United Kingdom with complete blood counts; thus, this is the largest study ever conducted on the hematological and associated parameters by degrees of animal food consumption. In the white population, 6 distinct diet groups were included, which allowed the comparison of characteristics across varying degrees of consumption of animal source foods, and we were also able to explore hematological indices separately in British Indians. As with all observational studies, some self-selection bias may be present, and only a modest proportion (5.5%) of invited participants agreed to take part, but a representative cohort is not necessary for making valid assessments of exposure–outcome associations (28). Because the study is cross-sectional, it was not possible to determine causality, and generalizability to other populations, especially ethnic groups other than white British or British Indians, might be limited. The findings would have been enhanced by the inclusion of various relevant indices, including serum ferritin, serum vitamin B-12, and other related markers, which were not available in this study. In addition, we did not determine the prevalence of genetic hemoglobin disorders, which can affect hemoglobin concentrations.

In conclusion, in this large population study in the United Kingdom, people with low or no red meat intake generally had lower hemoglobin concentrations and were slightly more likely to be anemic. In the white population, but not the British Indians, low or non-meat eaters also had lower white blood cell counts. Both white and British Indian vegetarians had higher mean platelet counts and lower mean platelet volumes compared with meat eaters, whereas white vegans had lower mean platelet counts but higher platelet volumes. Future studies should investigate the mechanisms that explain these differences because they might be related to chronic disease risk.

## Supplementary Material

nqz072_Supplemental_File

## Notes

This work was supported by the UK Medical Research Council (MR/M012190/1), Wellcome Trust Our Planet Our Health (Livestock, Environment and People, LEAP 205212/Z/16/Z), and Cancer Research UK (C8221/A19170). KEB is supported by the Girdlers’ New Zealand Health Research Council Fellowship. The funders had no role in the design of the study; in the collection, analyses, or interpretation of data; in the writing of the manuscript; or in the decision to publish the results.

The UK Biobank is an open-access resource. Bona fide researchers can apply to use the UK Biobank data set by registering and applying at http://www.ukbiobank.ac.uk/register-apply.

Supplemental Methods, Supplemental Figure 1, and Supplemental Tables 1–10 are available from the “Supplementary data” link in the online posting of the article and from the same link in the online table of contents at https://academic.oup.com/ajcn/.
