# Supplementary material for: Hematological parameters and prevalence of anemia in white and British Indian vegetarians and nonvegetarians in the UK Biobank
Source: Am J Clin Nutr. 2019 Jun 13;110(2):461–72. doi: 10.1093/ajcn/nqz072 (PMC6669054; doi:10.1093/ajcn/nqz072)
Supplement: nqz072_Supplemental_File [file nqz072_supplemental_file.docx]

**Hematological parameters and prevalence of anemia in white and British Indian vegetarians and nonvegetarians in the UK Biobank.**

Tammy YN Tong*^1^*, Timothy J Key*^1^*, Kezia Gaitskell*^1,2^*, Timothy J Green*^3^*, Wenji Guo*^1^*, Thomas A Sanders*^4^*, Kathryn E Bradbury*^1,5^*

1. Cancer Epidemiology Unit, Nuffield Department of Population Health, University of Oxford, Oxford, UK
2. Nuffield Division of Clinical Laboratory Sciences, Radcliffe Department of Medicine, University of Oxford, Oxford, UK
3. Discipline of Paediatrics and Reproductive Health, School of Medicine, University of Adelaide, Australia
4. Department of Nutritional Sciences, King’s College London, London, UK
5. National Institute for Health Innovation, School of Population Health, Faculty of Medical and Health Sciences, University of Auckland, Auckland, New Zealand

**Supplementary methods**

*Quality control procedures for blood count data*

Prior to any analyses, daily analyzer maintenance was carried out according to the manufacturer’s recommendations, and three manufacturers’ controls were run (1). Participant samples were only analyzed if the parameters for all the control material were sufficiently met (i.e. quality control check passed). Therefore, day-to-day variation of the assays throughout the recruitment period should be minimal.

Samples were typically assayed within 24 hours of blood draw. Within day variation of some white cell subtypes (mostly basophil and monocytes), but not total white cells, was observed. This might be due to the fact that cells could deteriorate as the samples age throughout the day, which might result in some misclassification of cells of same type, as these cells tend to cluster together in the analyzer output (1). However, there is no reason to suspect systematic differences in time from blood draw to assay across the diet groups, and therefore any such variation should be independent of diet group.

1. Sheard SM, Froggatt J. UK Biobank Haematology Data Companion Document [Internet]. 2017. Available from: https://biobank.ctsu.ox.ac.uk/crystal/docs/haematology.pdf

502,697 participants recruited and provided informed consent

Excluded 154 participants who subsequently requested to withdraw from the study

502,543 participants

Excluded 24,336 participants with no haematological data

478,207 participants

Excluded 21,851 participants of other or unknown ethnicities

450,780 white participants and 5,576 Indian participants

Excluded 3,054 white participants and 339 Indian participants who could not be classified into one of the prespecified diet groups

447,726 white participants and 5,237 Indian participants

**Supplementary figure 1**: Participant flow chart of the study

**Supplementary table 1**. Hemoglobin, red blood cell, and reticulocyte count in white British men by diet group in the UK Biobank (n=205,049).

| **Cell count***^1^* | **Meat eaters** | | | **Fish eaters**  **Max N=2,789** | **Vegetarians**  **Max N=2,130** | **Vegans**  **Max N=166** | ***p*-het*^3^*** |
| --- | --- | --- | --- | --- | --- | --- | --- |
|  | **Regular consumption**  **(>3 times/week)*^2^***  **Max N*^4^*=121,433** | **Low consumption**  **(≤3 times/week)*^2^***  **Max N=77,457** | **Poultry eaters**  **Max N=1,074** |  |  |  |  |
| **Mean Hemoglobin, g/L (95% CI)** | 150.3 (150.2, 150.3)^a^ | 149.8 (149.7, 149.9)^b^ | 148.5 (147.9, 149.1)^c^ | 147.8 (147.4, 148.1)^c^ | 147.6 (147.1, 148.0)^c^ | 144.8 (143.2, 146.3)^d^ | <0.001 |
| + smoking | 150.2 (150.2, 150.3)^a^ | 149.8 (149.7, 149.9)^b^ | 148.5 (147.9, 149.1)^c^ | 147.8 (147.4, 148.2)^c^ | 147.6 (147.2, 148.0)^c^ | 144.8 (143.3, 146.4)^d^ | <0.001 |
| **Mean red blood cell count, 10^12^ cells/L (95% CI)** | 4.74 (4.74, 4.74) ^a^ | 4.73 (4.73, 4.73) ^b^ | 4.69 (4.67, 4.72) ^c^ | 4.67 (4.66, 4.68) ^c^ | 4.67 (4.66, 4.69) ^c^ | 4.50 (4.45, 4.56) ^d^ | <0.001 |
| + smoking | 4.74 (4.74, 4.74) ^a^ | 4.73 (4.73, 4.73) ^b^ | 4.69 (4.67, 4.71) ^c^ | 4.67 (4.65, 4.68) ^c^ | 4.67 (4.66, 4.69) ^c^ | 4.50 (4.45, 4.56) ^d^ | <0.001 |
| **Mean reticulocyte, % (95% CI)** | 1.40 (1.40, 1.41) ^a^ | 1.34 (1.34, 1.35) ^b^ | 1.30 (1.24, 1.35) ^bc^ | 1.27 (1.23, 1.30) ^c^ | 1.27 (1.23, 1.31) ^c^ | 1.30 (1.16, 1.43) ^abc^ | <0.001 |
| + smoking | 1.40 (1.40, 1.41) ^a^ | 1.35 (1.34, 1.35) ^b^ | 1.30 (1.25, 1.36) ^bc^ | 1.26 (1.23, 1.30) ^c^ | 1.27 (1.23, 1.31) ^c^ | 1.29 (1.15, 1.42) ^abc^ | <0.001 |
| **Mean immature reticulocyte fraction, (95% CI)** | 0.29 (0.29, 0.29) ^a^ | 0.29 (0.29, 0.29) ^b^ | 0.28 (0.27, 0.28) ^c^ | 0.28 (0.28, 0.28) ^c^ | 0.28 (0.28, 0.28) ^c^ | 0.29 (0.28, 0.29) ^abc^ | <0.001 |
| + smoking | 0.29 (0.29, 0.29) ^a^ | 0.29 (0.29, 0.29) ^b^ | 0.28 (0.28, 0.28) ^c^ | 0.28 (0.28, 0.28) ^c^ | 0.28 (0.28, 0.28) ^c^ | 0.28 (0.28, 0.29) ^abc^ | <0.001 |

Abbreviations: CI=confidence interval. Groups that do not share a superscript letter were significantly different at the 5% level from post hoc pairwise comparisons based on linear regression models and after Bonferroni correction for multiple comparisons.
1. Results show adjusted mean levels (95% confidence intervals), estimated based on linear regression models. All estimates were adjusted for age at recruitment (<45, 45–49, 50–54, 55–59, 60–64, and ≥65 years), and additionally adjusted for smoking (never, previous, current <15 cigarettes/day, current 15 or more cigarettes/day, unknown) in subsequent row.
2. Includes participants who consume any red or processed meat (beef, lamb, pork, processed meat), regardless of whether they consume poultry, fish, or dairy. Cut-offs of regular and low consumption determined based on consumption of red and processed meat as reported on the touchscreen questionnaire.
3. Represents *p* for heterogeneity across the six diet groups, estimated by regressing each row variable against diet group.
4. Max N=maximum number, minimum number for these analyses (for mean immature reticulocyte fraction) included 119,499 regular meat eaters, 76,146 low meat eaters, 1,054 poultry eaters, 2,726 fish eaters, 2088 vegetarians, and 163 vegans.

**Supplementary table 2**. Hemoglobin, red blood cell, and reticulocyte count in white British premenopausal women by diet group in the UK Biobank (n=55,170).

| **Cell count***^1^* | **Meat eaters** | | | **Fish eaters**  **Max N=2,287** | **Vegetarians**  **Max N=1,638** | **Vegans**  **Max N=76** | ***p*-het*^3^*** |
| --- | --- | --- | --- | --- | --- | --- | --- |
|  | **Regular consumption**  **(>3 times/week)*^2^***  **Max N*^4^*=20,791** | **Low consumption**  **(≤3 times/week) *^2^***  **Max N=29,570** | **Poultry eaters**  **Max N=810** |  |  |  |  |
| **Mean Hemoglobin, g/L (95% CI)** | 133.0 (132.9, 133.2)^a^ | 132.3 (132.2, 132.4)^b^ | 130.5 (129.8, 131.2)^c^ | 130.1 (129.7, 130.5)^c^ | 130.6 (130.1, 131.1)^c^ | 131.4 (129.1, 133.8)^abc^ | <0.001 |
| + smoking | 133.0 (132.9, 133.2)^a^ | 132.3 (132.2, 132.4)^b^ | 130.4 (129.7, 131.1)^c^ | 130.2 (129.8, 130.6)^c^ | 130.7 (130.2, 131.2)^c^ | 131.5 (129.2, 133.8)^abc^ | <0.001 |
| **Mean red blood cell count, 10^12^ cells/L (95% CI)** | 4.28 (4.28, 4.29) ^a^ | 4.25 (4.24, 4.25) ^b^ | 4.19 (4.17, 4.22) ^c^ | 4.20 (4.19, 4.21) ^c^ | 4.22 (4.21, 4.24) ^c^ | 4.17 (4.09, 4.24) ^bc^ | <0.001 |
| + smoking | 4.28 (4.28, 4.29) ^a^ | 4.25 (4.24, 4.25) ^b^ | 4.20 (4.17, 4.22) ^c^ | 4.20 (4.19, 4.21) ^c^ | 4.22 (4.21, 4.24) ^c^ | 4.17 (4.10, 4.24) ^bc^ | <0.001 |
| **Mean reticulocyte, % (95% CI)** | 1.34 (1.33, 1.35) ^a^ | 1.30 (1.29, 1.31) ^b^ | 1.20 (1.14, 1.26) ^c^ | 1.21 (1.18, 1.25) ^c^ | 1.24 (1.20, 1.28) ^bc^ | 1.25 (1.05, 1.44) ^abc^ | <0.001 |
| + smoking | 1.34 (1.33, 1.35) ^a^ | 1.30 (1.29, 1.31) ^b^ | 1.20 (1.14, 1.26) ^c^ | 1.21 (1.18, 1.25) ^c^ | 1.24 (1.20, 1.28) ^bc^ | 1.24 (1.05, 1.44) ^abc^ | <0.001 |
| **Mean immature reticulocyte fraction, (95% CI)** | 0.29 (0.29, 0.29) ^a^ | 0.29 (0.28, 0.29) ^ab^ | 0.28 (0.28, 0.29) ^abc^ | 0.28 (0.28, 0.28) ^bc^ | 0.28 (0.28, 0.28) ^c^ | 0.28 (0.27, 0.30) ^abc^ | <0.001 |
| + smoking | 0.29 (0.29, 0.29) ^a^ | 0.29 (0.28, 0.29) ^ab^ | 0.28 (0.28, 0.29) ^abc^ | 0.28 (0.28, 0.28) ^bc^ | 0.28 (0.28, 0.28) ^c^ | 0.28 (0.27, 0.30) ^abc^ | <0.001 |

Abbreviations: CI=confidence interval. Groups that do not share a superscript letter were significantly different at the 5% level from post hoc pairwise comparisons based on linear regression models and after Bonferroni correction for multiple comparisons.
1. Results show adjusted mean levels (95% confidence intervals), estimated based on linear regression models. All estimates were adjusted for age at recruitment (<45, 45–49, 50–54, 55–59, 60–64, and ≥65 years), and additionally adjusted for smoking (never, previous, current <15 cigarettes/day, current 15 or more cigarettes/day, unknown) in subsequent row.
2. Includes participants who consume any red or processed meat (beef, lamb, pork, processed meat), regardless of whether they consume poultry, fish, or dairy. Cut-offs of regular and low consumption determined based on consumption of red and processed meat as reported on the touchscreen questionnaire.
3. Represents *p* for heterogeneity across the six diet groups, estimated by regressing each row variable against diet group.
4. Max N=maximum number, minimum number for these analyses (for mean immature reticulocyte fraction) included 20,463 regular meat eaters, 29,072 low meat eaters, 799 poultry eaters, 2,239 fish eaters, 1606 vegetarians, and 73 vegans.

**Supplementary table 3**. Hemoglobin, red blood cell, and reticulocyte count in white British postmenopausal women by diet group in the UK Biobank (n=178,501).

| **Cell count*^1^*** | **Meat eaters** | | | **Fish eaters**  **Max N=4,643** | **Vegetarians**  **Max N=2,583** | **Vegans**  **Max N=149** | ***p*-het*^3^*** |
| --- | --- | --- | --- | --- | --- | --- | --- |
|  | **Regular consumption**  **(>3 times/week)*^2^***  **Max N*^4^*=67,145** | **Low consumption**  **(≤3 times/week)*^2^***  **Max N=101,184** | **Poultry eaters**  **Max N=2,797** |  |  |  |  |
| **Mean Hemoglobin, g/L (95% CI)** | 136.6 (136.5, 136.6)^a^ | 135.8 (135.8, 135.9)^b^ | 134.2 (133.9, 134.5)^c^ | 133.9 (133.6, 134.1)^c^ | 133.7 (133.4, 134.1)^c^ | 134.7 (133.2, 136.1)^abc^ | <0.001 |
| + smoking | 136.5 (136.4, 136.6)^a^ | 135.8 (135.8, 135.9)^b^ | 134.3 (134.0, 134.6)^c^ | 134.0 (133.7, 134.2)^c^ | 133.8 (133.5, 134.2)^c^ | 134.8 (133.3, 136.2)^abc^ | <0.001 |
| **Mean red blood cell count, 10^12^ cells/L (95% CI)** | 4.36 (4.35, 4.36) ^a^ | 4.33 (4.33, 4.33) ^b^ | 4.27 (4.26, 4.28) ^c^ | 4.28 (4.27, 4.28) ^c^ | 4.29 (4.28, 4.31) ^c^ | 4.27 (4.21, 4.32) ^bc^ | <0.001 |
| + smoking | 4.36 (4.35, 4.36) ^a^ | 4.33 (4.33, 4.33) ^b^ | 4.27 (4.26, 4.28) ^c^ | 4.28 (4.27, 4.29) ^c^ | 4.29 (4.28, 4.31) ^c^ | 4.27 (4.21, 4.32) ^bc^ | <0.001 |
| **Mean reticulocyte, % (95% CI)** | 1.37 (1.36, 1.37) ^a^ | 1.31 (1.31, 1.32) ^b^ | 1.22 (1.19, 1.26) ^c^ | 1.20 (1.18, 1.23) ^c^ | 1.21 (1.18, 1.25) ^c^ | 1.23 (1.07, 1.38) ^abc^ | <0.001 |
| + smoking | 1.37 (1.36, 1.37) ^a^ | 1.31 (1.31, 1.32) ^b^ | 1.22 (1.18, 1.25) ^c^ | 1.20 (1.17, 1.23) ^c^ | 1.21 (1.18, 1.25) ^c^ | 1.22 (1.07, 1.38) ^abc^ | <0.001 |
| **Mean immature reticulocyte fraction, (95% CI)** | 0.29 (0.29, 0.30) ^a^ | 0.29 (0.29, 0.29) ^b^ | 0.28 (0.28, 0.28) ^c^ | 0.28 (0.28, 0.28) ^c^ | 0.28 (0.28, 0.28) ^c^ | 0.28 (0.27, 0.29) ^bc^ | <0.001 |
| + smoking | 0.29 (0.29, 0.29) ^a^ | 0.29 (0.29, 0.29) ^b^ | 0.28 (0.28, 0.28) ^c^ | 0.28 (0.28, 0.28) ^c^ | 0.28 (0.28, 0.28) ^c^ | 0.28 (0.27, 0.29) ^bc^ | <0.001 |

Abbreviations: CI=confidence interval. Groups that do not share a superscript letter were significantly different at the 5% level from post hoc pairwise comparisons based on linear regression models and after Bonferroni correction for multiple comparisons.
1. Results show adjusted mean levels (95% confidence intervals), estimated based on linear regression models. All estimates were adjusted for age at recruitment (<45, 45–49, 50–54, 55–59, 60–64, and ≥65 years), and additionally adjusted for smoking (never, previous, current <15 cigarettes/day, current 15 or more cigarettes/day, unknown) in subsequent row.
2. Includes participants who consume any red or processed meat (beef, lamb, pork, processed meat), regardless of whether they consume poultry, fish, or dairy. Cut-offs of regular and low consumption determined based on consumption of red and processed meat as reported on the touchscreen questionnaire.
3. Represents *p* for heterogeneity across the six diet groups, estimated by regressing each row variable against diet group.
4. Max N=maximum number, minimum number for these analyses (for mean immature reticulocyte fraction) included 66,067 regular meat eaters, 99,346 low meat eaters, 2,743 poultry eaters, 4,546 fish eaters, 2532 vegetarians, and 143 vegans.

**Supplementary table 4**. Hemoglobin, red blood cell, and reticulocyte count in British Indian men by diet group in the UK Biobank (n=2,739).

| **Cell count*^1^*** | **Meat eaters**  **Max N*^3^*=2,254** | **Vegetarians**  **Max N=485** | ***p*-het*^2^*** |
| --- | --- | --- | --- |
|  |  |  |  |
| **Mean Hemoglobin, g/L (95% CI)** | 147.2 (146.7, 147.7) | 145.2 (144.1, 146.2) | <0.001 |
| + smoking | 147.2 (146.7, 147.7) | 145.2 (144.2, 146.3) | <0.001 |
| **Mean red blood cell count, 10^12^ cells/L (95% CI)** | 4.92 (4.90, 4.94) | 4.91 (4.87, 4.95) | 0.77 |
| + smoking | 4.92 (4.90, 4.94) | 4.91 (4.87, 4.95) | 0.55 |
| **Mean reticulocyte, % (95% CI)** | 1.37 (1.34, 1.39) | 1.27 (1.22, 1.32) | <0.001 |
| + smoking | 1.37 (1.34, 1.39) | 1.27 (1.22, 1.32) | 0.001 |
| **Mean immature reticulocyte fraction, (95% CI)** | 0.30 (0.29, 0.30) | 0.29 (0.28, 0.29) | <0.001 |
| + smoking | 0.30 (0.29, 0.30) | 0.29 (0.28, 0.29) | <0.001 |

Abbreviations: CI=confidence interval.
1. Results show adjusted mean levels (95% confidence intervals), estimated based on linear regression models. All estimates were adjusted for age at recruitment (<45, 45–49, 50–54, 55–59, 60–64, and ≥65 years), and additionally adjusted for smoking (never, previous, current <15 cigarettes/day, current 15 or more cigarettes/day, unknown) in subsequent row.
2. Represents *p* for heterogeneity across the two diet groups, estimated by regressing each row variable against diet group.
3. Max N=maximum number, minimum number for these analyses (for mean immature reticulocyte fraction) included 2200 meat eaters and 467 vegetarians.

**Supplementary table 5**. Hemoglobin, red blood cell, and reticulocyte count in British Indian premenopausal women by diet group in the UK Biobank (n=794).

| **Cell count*^1^*** | **Meat eaters**  **Max N*^3^*=565** | **Vegetarians**  **Max N=229** | ***p*-het*^2^*** |
| --- | --- | --- | --- |
|  |  |  |  |
| **Mean Hemoglobin, g/L (95% CI)** | 127.8 (126.8, 128.7) | 125.1 (123.5, 126.6) | 0.004 |
| + smoking | 127.7 (126.7, 128.7) | 125.3 (123.7, 126.8) | 0.010 |
| **Mean red blood cell count, 10^12^ cells/L (95% CI)** | 4.44 (4.41, 4.47) | 4.42 (4.37, 4.47) | 0.47 |
| + smoking | 4.44 (4.41, 4.47) | 4.42 (4.37, 4.47) | 0.51 |
| **Mean reticulocyte, % (95% CI)** | 1.37 (1.32, 1.41) | 1.31 (1.24, 1.38) | 0.19 |
| + smoking | 1.36 (1.32, 1.41) | 1.31 (1.24, 1.38) | 0.23 |
| **Mean immature reticulocyte fraction, (95% CI)** | 0.30 (0.29, 0.30) | 0.30 (0.29, 0.31) | 0.46 |
| + smoking | 0.30 (0.29, 0.30) | 0.30 (0.29, 0.31) | 0.50 |

Abbreviations: CI=confidence interval.
1. Results show adjusted mean levels (95% confidence intervals), estimated based on linear regression models. All estimates were adjusted for age at recruitment (<45, 45–49, 50–54, 55–59, 60–64, and ≥65 years), and additionally adjusted for smoking (never, previous, current <15 cigarettes/day, current 15 or more cigarettes/day, unknown) in subsequent row.
2. Represents *p* for heterogeneity across the two diet groups, estimated by regressing each row variable against diet group.
3. Max N=maximum number, minimum number for these analyses (for mean immature reticulocyte fraction) included 550 meat eaters and 215 vegetarians.

**Supplementary table 6**. Hemoglobin, red blood cell, and reticulocyte count in British Indian postmenopausal women by diet group in the UK Biobank (n=1,595).

| **Cell count*^1^*** | **Meat eaters**  **Max N*^3^*=980** | **Vegetarians**  **Max N=615** | ***p*-het*^2^*** |
| --- | --- | --- | --- |
|  |  |  |  |
| **Mean Hemoglobin, g/L (95% CI)** | 130.6 (130.0, 131.2) | 127.9 (127.1, 128.7) | <0.001 |
| + smoking | 130.5 (129.9, 131.2) | 128.0 (127.2, 128.9) | <0.001 |
| **Mean red blood cell count, 10^12^ cells/L (95% CI)** | 4.45 (4.42, 4.47) | 4.42 (4.38, 4.45) | 0.13 |
| + smoking | 4.45 (4.42, 4.47) | 4.42 (4.38, 4.45) | 0.12 |
| **Mean reticulocyte, % (95% CI)** | 1.35 (1.31, 1.38) | 1.25 (1.20, 1.29) | <0.001 |
| + smoking | 1.35 (1.31, 1.38) | 1.25 (1.21, 1.29) | <0.001 |
| **Mean immature reticulocyte fraction, (95% CI)** | 0.30 (0.30, 0.30) | 0.29 (0.28, 0.29) | <0.001 |
| + smoking | 0.30 (0.30, 0.30) | 0.29 (0.28, 0.29) | <0.001 |

Abbreviations: CI=confidence interval.
1. Results show adjusted mean levels (95% confidence intervals), estimated based on linear regression models. All estimates were adjusted for age at recruitment (<45, 45–49, 50–54, 55–59, 60–64, and ≥65 years), and additionally adjusted for smoking (never, previous, current <15 cigarettes/day, current 15 or more cigarettes/day, unknown) in subsequent row.
2. Represents *p* for heterogeneity across the two diet groups, estimated by regressing each row variable against diet group.
3. Max N=maximum number, minimum number for these analyses (for mean immature reticulocyte fraction) included 951 meat eaters and 597 vegetarians.

**Supplementary table 7.** Anemia, low platelet count, or elevated platelet volume in white British participants by diet group in the UK Biobank (n=447,726).

| **Classification** | **Meat eaters** | | | **Fish eaters**  **Max N=10,042** | **Vegetarians**  **Max N=6,548** | **Vegans**  **Max N=398** | ***p*-het*^2^*** |
| --- | --- | --- | --- | --- | --- | --- | --- |
|  | **Regular consumption**  **(>3 times/week)*^1^***  **Max N=212,831** | **Low consumption**  **(≤3 times/week)*^1^***  **Max N=213,092** | **Poultry eaters**  **Max N=4,815** |  |  |  |  |
| **Anemia in men, number (%)*^3^*** | 3517 (2.9) ^a^ | 2200 (2.8) ^a^ | 43 (4.0) ^ab^ | 108 (3.9) ^b^ | 83 (3.9) ^ab^ | 11 (6.6) ^ab^ | <0.001 |
| Restricted to no iron supplement use | 3280 (2.8) ^a^ | 2053 (2.7) ^a^ | 40 (3.9) ^ab^ | 99 (3.8) ^b^ | 79 (4.0) ^b^ | 11 (7.3) ^b^ | <0.001 |
| Restricted to no B vitamins use | 3334 (2.8) ^a^ | 2097 (2.8) ^a^ | 40 (4.0) ^ab^ | 100 (3.8) ^b^ | 77 (3.9) ^ab^ | 6 (4.3) ^ab^ | <0.001 |
| Detailed correction for smoking***^4^*** | 3742 (3.1) ^ab^ | 2332 (3.0) ^a^ | 45 (4.2) ^ab^ | 113 (4.1) ^b^ | 88 (4.1) ^b^ | 11 (6.6) ^ab^ | <0.001 |
| **Anemia in premenopausal women, number (%)*^3^*** | 1804 (8.7) ^a^ | 2888 (9.8) ^b^ | 105 (13.0) ^c^ | 309 (13.5) ^c^ | 209 (12.8) ^c^ | 6 (7.9) ^abc^ | <0.001 |
| Restricted to no iron supplement use | 1625 (8.3) ^a^ | 2616 (9.4) ^b^ | 95 (12.9) ^c^ | 256 (12.9) ^c^ | 171 (12.2) ^c^ | 6 (8.7) ^abc^ | <0.001 |
| Restricted to no B vitamins use | 1734 (8.7) ^a^ | 2748 (9.8) ^b^ | 96 (12.9) ^bc^ | 288 (13.5) ^c^ | 197 (13.1) ^c^ | 3 (5.2) ^abc^ | <0.001 |
| Detailed correction for smoking***^4^*** | 1868 (9.0) ^a^ | 2996 (10.1) ^b^ | 112 (13.8) ^c^ | 316 (13.8) ^c^ | 215 (13.1) ^c^ | 6 (7.9) ^abc^ | <0.001 |
| **Anemia in postmenopausal women, number (%)*^3^*** | 2300 (3.4) ^a^ | 3838 (3.8) ^b^ | 154 (5.5) ^c^ | 248 (5.3) ^c^ | 149 (5.8) ^c^ | 6 (4.0) ^abc^ | <0.001 |
| Restricted to no iron supplement use | 2168 (3.3) ^a^ | 3634 (3.7) ^b^ | 141 (5.3) ^c^ | 225 (5.2) ^c^ | 137 (5.8) ^c^ | 5 (4.0) ^abc^ | <0.001 |
| Restricted to no B vitamins use | 2179 (3.4) ^a^ | 3612 (3.8) ^b^ | 135 (5.4) ^c^ | 221 (5.2) ^c^ | 135 (5.8) ^c^ | 5 (4.2) ^abc^ | <0.001 |
| Detailed correction for smoking***^4^*** | 2371 (3.5) ^a^ | 3999 (4.0) ^b^ | 158 (5.6) ^c^ | 257 (5.5) ^c^ | 156 (6.0) ^c^ | 6 (4.0) ^abc^ | <0.001 |
| **Microcytic anemia, number (%)*^5^*** |  |  |  |  |  |  |  |
| Men | 345 (0.3) ^a^ | 229 (0.3) ^a^ | 7 (0.7) ^a^ | 15 (0.5) ^a^ | 11 (0.5) ^a^ | 0 (0.0) ^a^ | 0.012 |
| Premenopausal women | 566 (2.7) ^a^ | 830 (2.8) ^a^ | 28 (3.5) ^a^ | 84 (3.7) ^a^ | 55 (3.4) ^a^ | 2 (2.6) ^a^ | 0.085 |
| Postmenopausal women | 213 (0.3) ^a^ | 352 (0.3) ^a^ | 8 (0.3) ^ab^ | 24 (0.5) ^ab^ | 18 (0.7) ^bc^ | 3 (2.0) ^c^ | <0.001 |
| **Macrocytic anemia, number (%)*^6^*** |  |  |  |  |  |  |  |
| Men | 280 (0.2) ^a^ | 116 (0.1) ^b^ | 5 (0.5) ^ab^ | 4 (0.1) ^ab^ | 4 (0.2) ^ab^ | 3 (1.8) ^c^ | <0.001 |
| Premenopausal women | 10 (0.0) ^a^ | 16 (0.1) ^a^ | 3 (0.4) ^b^ | 5 (0.2) ^b^ | 2 (0.1) ^ab^ | 0 (0.0) ^ab^ | <0.001 |
| Postmenopausal women | 95 (0.1) ^a^ | 122 (0.1) ^a^ | 3 (0.1) ^a^ | 13 (0.3) ^a^ | 5 (0.2) ^a^ | 0 (0.0) ^a^ | 0.072 |
| **Low platelet count, number (%)*^7^*** | 12168 (5.7) ^a^ | 10630 (5.0) ^b^ | 263 (5.5) ^ab^ | 482 (4.8) ^bc^ | 264 (4.0) ^c^ | 35 (8.8) ^a^ | <0.001 |
| **Elevated platelet volume, number (%)*^8^*** | 10658 (5.0) ^a^ | 11513 (5.4) ^b^ | 281 (5.8) ^ab^ | 588 (5.9) ^b^ | 324 (4.9) ^ab^ | 51 (12.8) ^c^ | <0.001 |
| **Low platelet count & elevated volume, number (%)*^6,7^*** | 3046 (1.4) ^a^ | 2979 (1.4) ^b^ | 70 (1.5) ^ab^ | 152 (1.5) ^ab^ | 87 (1.3) ^c^ | 15 (3.8) ^d^ | <0.001 |

Abbreviations: MCV = mean corpuscular volume. Groups that do not share a superscript letter were significantly different at the 5% level from post hoc pairwise comparisons based on linear regression models and after Bonferroni correction for multiple comparisons.

1. Includes participants who consume any red or processed meat (beef, lamb, pork, processed meat), regardless of whether they consume poultry, fish, or dairy. Cut-offs of regular and low consumption determined based on consumption of red and processed meat as reported on the touchscreen questionnaire.
2. Represents *p* for heterogeneity across the six diet groups, estimated by regressing each row variable against diet group.
3. Anemia defined as Hemoglobin < 130 g/L for men and <120 g/L for women. For defining anemia, Hemoglobin is adjusted by -3 g/L in current smokers.
4. <10 cigarettes smoked per day = no adjustment; >=10 and <20 cigarettes smoked per day = -3 g/L; >=20 and <40 cigarettes smoked per day = -5 g/L; >=40 cigarettes smoked per day = -7 g/L; unknown amount = -3 g/L.
5. Microcytic anemia defined as Hemoglobin <130 g/L for men and <120 g/L for women, and MCV < 80 fL (both sexes).
6. Macrocytic anemia defined as Hemoglobin <130 g/L for men and <120 g/L for women, and MCV > 100 fL (both sexes).
7. Low platelet count defined as < 169.06 x 109 cells/L, as specified by the manufacturer reference range.
8. Elevated platelet volume defined as >11.24 fL, as specified by the manufacturer reference range.

**Supplementary table 8**. Anemia, low platelet count, or elevated platelet volume in British Indian participants by diet group in the UK Biobank (n=5,237).

| **Classification** | **Meat eaters**  **Max N=3,875** | **Vegetarians**  **Max N=1,362** | ***p*-het*^1^*** |
| --- | --- | --- | --- |
|  |  |  |  |
| **Anemia in men, number (%)*^2^*** | 172 (7.6) | 61 (12.6) | <0.001 |
| Restricted to no iron supplement use | 153 (7.0) | 55 (11.9) | <0.001 |
| Restricted to no B vitamins use | 160 (7.4) | 57 (12.3) | <0.001 |
| Detailed correction for smoking***^3^*** | 177 (7.8) | 62 (12.8) | <0.001 |
| **Anemia in premenopausal women, number (%)*^2^*** | 116 (20.5) | 61 (26.6) | 0.061 |
| Restricted to no iron supplement use | 93 (19.1) | 48 (26.2) | 0.045 |
| Restricted to no B vitamins use | 112 (21.3) | 56 (26.2) | 0.15 |
| Detailed correction for smoking***^3^*** | 116 (20.5) | 61 (26.6) | 0.061 |
| **Anemia in postmenopausal women, number (%)*^2^*** | 130 (13.3) | 118 (19.2) | 0.002 |
| Restricted to no iron supplement use | 107 (11.9) | 99 (18.2) | <0.001 |
| Restricted to no B vitamins use | 122 (13.3) | 105 (18.5) | 0.006 |
| Detailed correction for smoking***^3^*** | 131 (13.4) | 118 (19.2) | 0.002 |
| **Microcytic anemia, number (%)*^3^*** |  |  |  |
| Men | 66 (2.9) | 22 (4.5) | 0.069 |
| Premenopausal women | 58 (10.3) | 36 (15.7) | 0.031 |
| Postmenopausal women | 45 (4.6) | 35 (5.7) | 0.33 |
| **Macrocytic anemia, number (%)*^4^*** |  |  |  |
| Men | 4 (0.2) | 2 (0.4) | 0.32 |
| Premenopausal women | 1 (0.2) | 0 (0.0) | 0.52 |
| Postmenopausal women | 1 (0.1) | 2 (0.3) | 0.32 |
| **Low platelet count, number (%)*^5^*** | 225 (5.8) | 43 (3.2) | <0.001 |
| **Elevated platelet volume, number (%)*^6^*** | 264 (6.8) | 70 (5.1) | 0.030 |
| **Low platelet count & elevated volume, number (%)*^5,6^*** | 84 (2.2) | 11 (0.8) | 0.001 |

Abbreviations: MCV = mean corpuscular volume.

1. Represents *p* for heterogeneity across the six diet groups, estimated by regressing each row variable against diet group.
2. Anemia defined as Hemoglobin < 130 g/L for men and <120 g/L for women. For defining anemia, Hemoglobin is adjusted by -3 g/L in current smokers.
3. <10 cigarettes smoked per day = no adjustment; >=10 and <20 cigarettes smoked per day = -3 g/L; >=20 and <40 cigarettes smoked per day = -5 g/L; >=40 cigarettes smoked per day = -7 g/L; unknown amount = -3 g/L.
4. Microcytic anemia defined as Hemoglobin <130 g/L for men and <120 g/L for women, and MCV < 80 fL (both sexes).
5. Macrocytic anemia defined as Hemoglobin <130 g/L for men and <120 g/L for women, and MCV > 100 fL (both sexes).
6. Low platelet count defined as < 169.06 x 109 cells/L, as specified by the manufacturer reference range.
7. Elevated platelet volume defined as >11.24 fL, as specified by the manufacturer reference range.

**Supplementary table 9**.White blood cell and platelet count in white British participants by diet group in the UK Biobank (n=447,726).

| **Mean cell count or volume, 95% CI*^1^*** | **Meat eaters** | | | **Fish eaters**  **Max N=10,042** | **Vegetarians**  **Max N=6,548** | **Vegans**  **Max N=398** | ***p*-het*^3^*** |
| --- | --- | --- | --- | --- | --- | --- | --- |
|  | **Regular consumption**  **(>3 times/week)*^1^***  **Max N*^4^*=212,831** | **Low consumption**  **(≤3 times/week)*^1^***  **Max N=213,092** | **Poultry eaters**  **Max N=4,815** |  |  |  |  |
| **White cell count, 10^9^ cells/L** | 7.02 (7.01, 7.03) ^a^ | 6.80 (6.79, 6.81) ^b^ | 6.55 (6.49, 6.61) ^c^ | 6.48 (6.44, 6.52) ^cd^ | 6.69 (6.63, 6.74) ^e^ | 6.22 (6.01, 6.43) ^d^ | <0.001 |
| + smoking | 7.00 (6.99, 7.01) ^a^ | 6.82 (6.81, 6.83) ^b^ | 6.58 (6.52, 6.64) ^c^ | 6.52 (6.48, 6.56) ^cd^ | 6.73 (6.68, 6.78) ^e^ | 6.26 (6.05, 6.46) ^d^ | <0.001 |
| + smoking, no illness only | 6.84 (6.83, 6.85) ^a^ | 6.68 (6.67, 6.69) ^b^ | 6.47 (6.41, 6.53) ^c^ | 6.43 (6.40, 6.47) ^c^ | 6.60 (6.55, 6.65) ^d^ | 6.18 (5.97, 6.39) ^c^ | <0.001 |
| **Neutrophils count, 10^9^ cells/L** | 4.34 (4.33, 4.34) ^a^ | 4.18 (4.17, 4.18) ^b^ | 4.04 (4.00, 4.08) ^c^ | 3.96 (3.93, 3.99) ^d^ | 4.18 (4.14, 4.21) ^b^ | 3.96 (3.82, 4.10) ^cd^ | <0.001 |
| + smoking | 4.32 (4.32, 4.33) ^a^ | 4.19 (4.18, 4.20) ^b^ | 4.06 (4.03, 4.10) ^c^ | 3.99 (3.96, 4.02) ^d^ | 4.21 (4.17, 4.24) ^b^ | 3.98 (3.85, 4.12) ^cd^ | <0.001 |
| + smoking, no illness only | 4.20 (4.19, 4.21) ^a^ | 4.09 (4.08, 4.09) ^b^ | 3.97 (3.92, 4.01) ^c^ | 3.92 (3.89, 3.95) ^c^ | 4.13 (4.09, 4.16) ^a^ | 3.90 (3.74, 4.07) ^bc^ | <0.001 |
| **Lymphocyte count, 10^9^ cells/L** | 1.98 (1.97, 1.98) ^a^ | 1.94 (1.94, 1.95) ^b^ | 1.85 (1.82, 1.89) ^c^ | 1.85 (1.83, 1.87) ^c^ | 1.84 (1.81, 1.87) ^c^ | 1.65 (1.53, 1.76) ^d^ | <0.001 |
| + smoking | 1.97 (1.97, 1.98) ^a^ | 1.95 (1.94, 1.95) ^b^ | 1.86 (1.83, 1.89) ^c^ | 1.86 (1.84, 1.88) ^c^ | 1.85 (1.82, 1.88) ^c^ | 1.66 (1.54, 1.77) ^d^ | <0.001 |
| + smoking, no illness only | 1.95 (1.95, 1.96) ^a^ | 1.93 (1.93, 1.93) ^b^ | 1.86 (1.83, 1.89) ^cd^ | 1.86 (1.84, 1.88) ^c^ | 1.82 (1.80, 1.84) ^d^ | 1.66 (1.57, 1.76) ^e^ | <0.001 |
| **Monocyte count, 10^9^ cells/L** | 0.49 (0.48, 0.49) ^a^ | 0.47 (0.47, 0.47) ^b^ | 0.46 (0.45, 0.46) ^c^ | 0.46 (0.46, 0.47) ^c^ | 0.46 (0.45, 0.47) ^c^ | 0.44 (0.41, 0.46) ^bc^ | <0.001 |
| + smoking | 0.49 (0.48, 0.49) ^a^ | 0.47 (0.47, 0.47) ^b^ | 0.46 (0.45, 0.47) ^c^ | 0.46 (0.46, 0.47) ^c^ | 0.46 (0.45, 0.47) ^c^ | 0.44 (0.41, 0.46) ^bc^ | <0.001 |
| + smoking, no illness only | 0.47 (0.47, 0.47) ^a^ | 0.46 (0.46, 0.46) ^b^ | 0.45 (0.44, 0.46) ^c^ | 0.45 (0.45, 0.46) ^c^ | 0.45 (0.45, 0.46) ^c^ | 0.44 (0.42, 0.47) ^abc^ | <0.001 |
| **Eosinophil count, 10^9^ cells/L** | 0.18 (0.18, 0.18) ^a^ | 0.17 (0.17, 0.17) ^b^ | 0.16 (0.16, 0.17) ^cd^ | 0.17 (0.16, 0.17) ^bc^ | 0.17 (0.16, 0.17) ^bc^ | 0.14 (0.13, 0.16) ^d^ | <0.001 |
| + smoking | 0.18 (0.18, 0.18) ^a^ | 0.17 (0.17, 0.17) ^b^ | 0.16 (0.16, 0.17) ^cd^ | 0.17 (0.17, 0.17) ^bc^ | 0.17 (0.17, 0.17) ^bc^ | 0.15 (0.13, 0.16) ^d^ | <0.001 |
| + smoking, no illness only | 0.17 (0.17, 0.17) ^a^ | 0.17 (0.17, 0.17) ^b^ | 0.16 (0.15, 0.16) ^c^ | 0.16 (0.16, 0.17) ^bc^ | 0.16 (0.16, 0.17) ^bc^ | 0.14 (0.12, 0.16) ^c^ | <0.001 |
| **Basophil count, 10^9^ cells/L** | 0.04 (0.04, 0.04) ^a^ | 0.04 (0.04, 0.04) ^ab^ | 0.04 (0.04, 0.04) ^ab^ | 0.04 (0.04, 0.04) ^b^ | 0.04 (0.04, 0.04) ^a^ | 0.04 (0.03, 0.04) ^ab^ | <0.001 |
| + smoking | 0.04 (0.04, 0.04) ^ab^ | 0.04 (0.04, 0.04) ^a^ | 0.04 (0.04, 0.04) ^ab^ | 0.04 (0.04, 0.04) ^a^ | 0.04 (0.04, 0.04) ^b^ | 0.04 (0.03, 0.04) ^ab^ | <0.001 |
| + smoking, no illness only | 0.04 (0.04, 0.04) ^a^ | 0.04 (0.04, 0.04) ^b^ | 0.04 (0.04, 0.04) ^ab^ | 0.04 (0.04, 0.04) ^ab^ | 0.04 (0.04, 0.04) ^ab^ | 0.04 (0.03, 0.04) ^ab^ | <0.001 |
| **Platelet count, 10^9^ cells/L** | 254.5 (254.2, 254.7)^a^ | 252.1 (251.8, 252.3)^b^ | 248.2 (246.6, 249.9)^c^ | 250.7 (249.6, 251.9)^bc^ | 258.2 (256.8, 259.7)^d^ | 238.2 (232.5, 243.9)^e^ | <0.001 |
| + smoking | 254.4 (254.1, 254.6)^a^ | 252.2 (251.9, 252.4)^b^ | 248.4 (246.7, 250.0)^c^ | 250.9 (249.7, 252.0)^bc^ | 258.4 (257.0, 259.9)^d^ | 238.3 (232.6, 244.0)^e^ | <0.001 |
| + smoking, no illness only | 253.9 (253.6, 254.2)^a^ | 251.9 (251.6, 252.2)^b^ | 248.5 (246.5, 250.4)^cd^ | 250.7 (249.4, 252.0)^bc^ | 258.8 (257.2, 260.4)^e^ | 238.1 (231.2, 245.1)^d^ | <0.001 |
| **Platelet volume, fL** | 9.31 (9.31, 9.32) ^a^ | 9.34 (9.33, 9.34) ^b^ | 9.40 (9.37, 9.43) ^c^ | 9.34 (9.32, 9.36) ^ab^ | 9.27 (9.24, 9.29) ^d^ | 9.73 (9.63, 9.84) ^e^ | <0.001 |
| + smoking | 9.31 (9.31, 9.31) ^a^ | 9.34 (9.33, 9.34) ^b^ | 9.40 (9.37, 9.43) ^c^ | 9.34 (9.32, 9.36) ^b^ | 9.27 (9.24, 9.29) ^d^ | 9.73 (9.63, 9.84) ^e^ | <0.001 |
| + smoking, no illness only | 9.31 (9.31, 9.32) ^a^ | 9.34 (9.34, 9.35) ^b^ | 9.41 (9.37, 9.45) ^c^ | 9.38 (9.35, 9.40) ^bc^ | 9.28 (9.25, 9.31) ^a^ | 9.78 (9.65, 9.91) ^d^ | <0.001 |

Abbreviations: CI=confidence interval. Groups that do not share a superscript letter were significantly different at the 5% level from post hoc pairwise comparisons based on linear regression models and after Bonferroni correction for multiple comparisons.
1. Results show adjusted mean levels (95% confidence intervals), estimated based on linear regression models. All estimates were adjusted for age at recruitment (<45, 45–49, 50–54, 55–59, 60–64, and ≥65 years) and sex; or additionally adjusted for smoking (never, previous, current <15 cigarettes/day, current 15 or more cigarettes/day, unknown); and restricted to participants who reported no long-standing illness, disability, or infirmity (135,926 regular meat eaters, 144,499 low meat eaters, 3,219 poultry eaters, 7,136 fish eaters, 4,559 vegetarians, and 251 vegans) in subsequent rows.
2. Includes participants who consume any red or processed meat (beef, lamb, pork, processed meat), regardless of whether they consume poultry, fish, or dairy. Cut-offs of regular and low consumption determined based on consumption of red and processed meat as reported on the touchscreen questionnaire.
3. Represents *p* for heterogeneity across the six diet groups, estimated by regressing each row variable against diet group.
4. Max N=maximum number, minimum number for these analyses (for neutrophil, lymphocyte, monocyte, eosinophil, and basophil count) included 212,458 regular meat eaters, 212,708 low meat eaters, 4,805 poultry eaters, 10,017 fish eaters, 6,535 vegetarians, and 397 vegans.

**Supplementary table 10**.White blood cell and platelet count in British Indian participants by diet group in the UK Biobank (n=5,237).

| **Mean cell count or volume, 95% CI*^1^*** | **Meat eaters**  **Max N*^3^*=3,875** | **Vegetarians**  **Max N=1,362** | ***p*-het*^2^*** |
| --- | --- | --- | --- |
|  |  |  |  |
| **White cell count, 10^9^ cells/L** | 7.34 (7.29, 7.40) | 7.30 (7.21, 7.39) | 0.49 |
| + smoking | 7.33 (7.27, 7.38) | 7.34 (7.24, 7.43) | 0.89 |
| + smoking, no illness only | 7.27 (7.21, 7.34) | 7.24 (7.13, 7.35) | 0.64 |
| **Neutrophils count, 10^9^ cells/L** | 4.31 (4.26, 4.35) | 4.30 (4.22, 4.37) | 0.85 |
| + smoking | 4.30 (4.25, 4.34) | 4.32 (4.25, 4.39) | 0.54 |
| + smoking, no illness only | 4.25 (4.20, 4.30) | 4.22 (4.13, 4.30) | 0.54 |
| **Lymphocyte count, 10^9^ cells/L** | 2.29 (2.26, 2.31) | 2.26 (2.22, 2.30) | 0.21 |
| + smoking | 2.28 (2.26, 2.31) | 2.26 (2.22, 2.30) | 0.40 |
| + smoking, no illness only | 2.29 (2.26, 2.31) | 2.28 (2.24, 2.33) | 0.82 |
| **Monocyte count, 10^9^ cells/L** | 0.47 (0.47, 0.48) | 0.47 (0.46, 0.48) | 0.17 |
| + smoking | 0.47 (0.47, 0.48) | 0.47 (0.46, 0.48) | 0.23 |
| + smoking, no illness only | 0.47 (0.46, 0.48) | 0.46 (0.45, 0.47) | 0.23 |
| **Eosinophil count, 10^9^ cells/L** | 0.23 (0.22, 0.24) | 0.23 (0.22, 0.24) | 0.96 |
| + smoking | 0.23 (0.22, 0.24) | 0.23 (0.22, 0.24) | 0.80 |
| + smoking, no illness only | 0.22 (0.22, 0.23) | 0.23 (0.21, 0.24) | 0.82 |
| **Basophil count, 10^9^ cells/L** | 0.04 (0.04, 0.04) | 0.04 (0.04, 0.05) | 0.33 |
| + smoking | 0.04 (0.04, 0.04) | 0.04 (0.04, 0.05) | 0.22 |
| + smoking, no illness only | 0.04 (0.04, 0.04) | 0.04 (0.04, 0.05) | 0.19 |
| **Platelet count, 10^9^ cells/L** | 256.1 (254.1, 258.1) | 266.7 (263.3, 270.0) | <0.001 |
| + smoking | 256.1 (254.1, 258.1) | 266.7 (263.3, 270.1) | <0.001 |
| + smoking, no illness only | 256.3 (253.9, 258.6) | 265.3 (261.3, 269.2) | <0.001 |
| **Platelet volume, fL** | 9.39 (9.36, 9.43) | 9.24 (9.18, 9.30) | <0.001 |
| + smoking | 9.39 (9.36, 9.43) | 9.24 (9.18, 9.30) | <0.001 |
| + smoking, no illness only | 9.41 (9.36, 9.45) | 9.25 (9.17, 9.32) | <0.001 |

Abbreviations: CI=confidence interval.
1. Results show adjusted mean levels (95% confidence intervals), estimated based on linear regression models. All estimates were adjusted for age at recruitment (<45, 45–49, 50–54, 55–59, 60–64, and ≥65 years) and sex; or additionally adjusted for smoking (never, previous, current <15 cigarettes/day, current 15 or more cigarettes/day, unknown); and restricted to participants who reported no long-standing illness, disability, or infirmity (2,545 meat eaters, 920 vegetarians) in subsequent rows.
2. Represents *p* for heterogeneity across the two diet groups, estimated by regressing each row variable against diet group.
3. Max N=maximum number, minimum number for these analyses (for neutrophil, lymphocyte, monocyte, eosinophil, and basophil count) included 3,865 meat eaters and 1357 vegetarians.
